# The role of motor inhibition in implicit negation processing: two Go/No-Go behavioral studies

**DOI:** 10.1007/s00426-024-01941-0

**Published:** 2024-03-14

**Authors:** Martina Montalti, Marta Calbi, Maria Alessandra Umiltà, Vittorio Gallese, Valentina Cuccio

**Affiliations:** 1 Department of Medicine and Surgery, Unit of Neuroscience, University of Parma, Parma, Italy; 2 Lab Neuroscience & Humanities, University of Parma, Parma, Italy; 3 Department of Clinical and Experimental Sciences, University of Brescia, Brescia, Italy; 4 Department of Philosophy “Piero Martinetti”, State University of Milan, Milan, MI Italy; 5 Department of Food and Drug, University of Parma, Parma, Italy; 6 Department of Ancient and Modern Civilizations, University of Messina, Messina, Italy; 7 Department of Cognitive Sciences, Psychology, Education and Cultural Studies, University of Messina, Messina, Italy

## Abstract

Several studies demonstrated that explicit forms of negation processing (e.g., “I don’t know”) recruits motor inhibitory mechanisms. However, whether this is also true for implicit negation, in which the negative meaning is implicated but not explicitly lexicalized in the sentence (e.g., “I ignore”), has never been studied before. Two Go/No-Go studies, which differed only for the time-windows to respond to the Go stimulus, were carried out. In each, participants (*N* = 86 in experiment 1; *N* = 87 in experiment 2) respond to coloured circle while reading task-irrelevant affirmative, explicit negative and implicit negative sentences. We aimed to investigate whether: (i) the processing of implicit negations recruits inhibitory mechanisms; (ii) these inhibitory resources are differently modulated by implicit and explicit negations. Results show that implicit negative sentences recruit the inhibitory resources more strongly when compared to explicit ones, probably due to their inferential nature, likely requiring deeper processing of the negative meaning. Implicit and inferential meaning (i.e., pragmatic information) are grounded too in the same mechanisms that integrate action with perception. Such findings provide further evidence to the embodied account of language, showing that even abstract aspects, like implicit negation, are grounded in the sensory-motor system, by means of functional link between language and motor activity.

**Supplementary Information:**

The online version contains supplementary material available at 10.1007/s00426-024-01941-0.

## Introduction

Negation is a unique feature of human language, universally present in all natural languages and with no equal in any animal communication system (Speranza & Horn, 2010). Negation allows us to reverse the truth value of a sentence (Horn, 1989), i.e., the property of a sentence to be true or false, determining a semantic opposition between a negated expression and its positive counterparts. Many of our distinctive human practices, such as dealing with mathematical reasoning, arguing about philosophical hypotheses, or developing counterfactual reasoning (i.e., the tendency to imagine scenarios that could have taken place but did not occur), would not be possible without the use of negation. Even ethics and law would not be possible without negation since following moral and juridical rules implies the capacity to discern between what we ought to do and what we ought not. For these reasons, negation is a central topic in several fields. Within the sciences of language, negation has been the object of investigation at all linguistic levels. Indeed, morphosyntactic, syntactic, semantic, and pragmatic aspects of negation have been widely investigated (Horn, 2001). Within these studies, pragmatics certainly offers a privileged perspective for the understanding of the mechanisms underlying this logic operator since it focuses on the use of negation in context. In other words, a usage-based pragmatic approach allows us to ground the use of this operator in the dimension of human communication processes, thus relating the functioning of negation to the socio-cognitive mechanisms underlying it (Cuccio, 2011, 2012). Furthermore, a pragmatic investigation of negation also allows us to advance and deepen our knowledge of how negation works at all the other language levels. For example, based on a pragmatic perspective, the definition of negation as a semantic opposition (see above) has been reconsidered since negative statements are communicatively much more than inverted assertions and imply a different inferential impact, for example, on quantifiers or scalar words with respect to affirmative sentences.

In the last decades, the cognitive processes underlying negation and its neural underpinnings have also been object of investigation. As for the former, it has been shown that the processing of negation is cognitively more demanding compared to the processing of affirmative sentences, as reflected in higher error rates and longer reaction times (RTs). The cognitive load associated with the processing of sentential negation has been explained by cognitive effects such as reduced accessibility of the negated concept (Kaup, 2001; Kaup & Zwaan, 2003; MacDonald & Just, 1989), the elicitation of a complementary scenario (Kaup et al., 2005; Orenes et al., 2014) and the increase in cognitive effort (Carpenter & Just, 1975; Chase & Clark, 1972; Kaup et al., 2006). There is evidence that processing of polarity (i.e., defining whether a sentence is in the affirmative or negative form) in hand action-related sentences modulates the hand motor-related areas (Alemanno et al., 2012; Aravena et al., 2012; Bartoli et al., 2013; Foroni & Semin, 2013; Liuzza et al., 2011; Tettamanti et al., 2008; Tomasino et al., 2010). More recently, it has been proposed that processing linguistic negation might recruit the neural mechanisms underlying motor response inhibition (Beltrán et al., 2018, 2019; De Vega et al., 2016; Foroni & Semin, 2013; García-Marco et al., 2019; Liu et al., 2019, 2020; Montalti et al., 2021; Papeo et al., 2016; Vitale et al., 2022). This hypothesis is coherent with an embodied account of language, which posits that the comprehension of language is grounded in our sensory-motor system (Cuccio & Gallese, 2018; Di Cesare et al., 2017; Gallese, 2008; Gallese & Cuccio, 2018). Indeed, the functional links between language and motor activity have been widely investigated in recent years, and a vast amount of experimental data has corroborated the hypothesis that the same mechanisms that integrate action and perception may also play a crucial role in the processing of different types of linguistic information (Barsalou, 2010; Cuccio et al., 2014; Fischer & Zwaan, 2008; Gallese & Lakoff, 2005; Glenberg and Gallese, 2012; Glenberg et al., 2013; Jirak et al., 2010; Spadacenta et al., 2014; Mirabella et al., 2012/2017; Pulvermüller et al., 2014). In this framework, findings in support of the hypothesis that the processing of linguistic negation shares resources with motor inhibition have also been provided. For example, de Vega and colleagues (2016) carried out an electroencephalographic (EEG) study in which participants were asked to read negative and affirmative action-related sentences while performing a Go/No-Go task. This study showed that negative sentences modulate theta bands, a marker of motor inhibition, over the frontal cortex. In another EEG study, Beltrán et al. (2018) presented participants with negative and affirmative action-related sentences while they were performing a Stop-Signal Task (SST; Logan et al., 1984), which is used to evaluate reactive inhibition. Results showed that two event-related potentials (ERPs; N1 and P3) were enhanced by successful inhibition. Furthermore, these findings also showed that N1 amplitude was higher for negative sentences compared to affirmative ones in successful stop trials. Via source analysis, the authors suggested that N1 modulation depended on the right inferior frontal gyrus (rIFG), an area known to play a key role in inhibitory control (Aron et al., 2014).

Notably, experimental research on the cognitive and neural underpinnings of linguistic negation processing is mainly focused on sentential negation, i.e., forms of negation explicitly lexicalized at the sentence level using morpho-syntactic expressions, such as “not”, “no” or “don’t” which overtly convey a negative meaning. Few studies have been conducted on other forms of negation that rely more heavily on the pragmatic dimension (e.g., Xiang et al., 2016; Marrero et al., 2020). Particularly interesting in this regard is the distinction proposed by Clark (1976; but see also Horn, 1996) between explicit and implicit negation. While the latter represents a non-asserted negative meaning, explicit negation is present at the level of the asserted meaning of a sentence. Examples of explicit negation include not only words such as “no”, “not” or “don’t”, but also expressions like “few” and “little”, as well as prefixes such as im-moral or a-symmetry. In other words, explicit forms of negation encompass both morphosyntactic elements that directly convey a negative meaning, and expressions that, through entailments, result in the representation of a negative meaning in the minimal sentence. By contrast, implicit negation conveys a negative meaning that is not explicitly present in the minimal sentence, but rather in its intended meaning, relying on presuppositions or implicatures. According to Clark (1976), example of implicit negation are verbs such as “forget”, “prevent”, “avoid”, etc. As Clark states (1976, 1313) “[…] words as absent, forget, except, and without [.] are approximately synonymous with expressions that are considered negative, i.e., not present, not remember, but not, and not with respectively”. To clearly distinguish between explicit and implicit negation, Clark further explains that “in short, explicit negatives actually deny positive suppositions on the part of the speaker or listener (No, it isn’t true. Few men left.), while implicit negatives merely affirm the already negative suppositions of the speaker or listener (Yes, it’s true. A few men left.). In this sense, the explicit negatives really do deny, while the implicit negatives actually affirm” Clark, 1976, 1314). Thus, from a pragmatic perspective, explicit and implicit negation primarily differ in terms of the presuppositions they refer to. Explicit negation denies an affirmative presupposition whereas implicit negation confirms a negative presupposition. To clarify this point, we might recall the classic distinction between explicit and implicit meaning of a sentence, which dates to Grice (1989). According to Grice (1989), within each sentence, we can identify the level of what is literally said and the level of what is intended or implicated by that sentence. The construction of the sentence meaning i.e., its explicit meaning, relies on inferences known as entailments, which are directly derived from the literal meaning of the sentence components. On the other hand, the construction of the intended meaning relies on presuppositions, i.e., assumptions implicitly assumed by the speakers, as well as contextually based inferences known as implicatures. We will not delve into the debate on the inferential processes underlying both levels of sentences representation (for a discussion, see Carapezza & Cuccio, 2018). It suffices to say that there is no agreement on the nature of such processes. However, the distinction between explicit and implicit meaning can be considered one of the defining features of any pragmatic account of language.

The present study addresses the issue of the grounding of linguistic implicit negation in the mechanisms of motor inhibition. The study aims to determine whether the processing of sentences formulated in the affirmative form but containing implicit negation (e.g., the Italian verbs *digiunare*, *tacere*, *vietare*, *ignorare*, *rifiutare -* to fast, to shut up, to forbid, to ignore, and to refuse-) recruits the mechanisms of motor response inhibition, as it is the case for explicit negative sentences. While some studies have been carried out on the processing of implicit negation (Jones, 1968; Clark, 1976), this is the first study focusing on implicit negation under the hypothesis of the reuse of inhibitory resources. To accomplish this aim, we chose to employ the Go/No-go paradigm, for main two reasons. The first one is theoretical, as the Go/No-go task is undoubtedly one of the most widespread paradigms used to study motor control, and also to investigate the involvement of inhibitory mechanisms in sentence negation processing. The second one is practical, as the Go/No-go was more suitable for the online administration comparing to other more complex paradigms (e.g., the SST; Logan et al., 1984). Thus, the Go/No-go paradigm has been widely used in studies examining inhibitory mechanisms in the processing of sentence negation and it aligned well with the brevity and ease of completion required for an online study.

We predict an involvement of resources for motor inhibition during the processing of both explicit and implicit negative sentences compared to affirmative ones. Regarding the involvement of the motor inhibitory system in the two different types of negation, several scenarios could be expected. One possibility is that, since both explicit and implicit negative sentences contain negation, they recruit the motor inhibitory system similarly. Alternatively, a gradient effect might be observed, structured in two different ways. In the first case, explicit negation, being explicitly lexicalized in the sentence, might lead to a higher involvement of the motor inhibitory mechanisms compared to implicit negation. In the second case, implicit negation might lead to a higher activation of inhibitory resources, likely due to its inferential nature. Since implicit negation confirms a negative presupposition, the negation is not lexicalized in the minimal sentence but only presupposed. This may result in a deeper processing of the negative meaning compared to a potentially shallow processing of explicit negation. Indeed, implicit negation might be processed at a pragmatic level compared to the semantic processing of the negative meaning which takes place in explicit negative sentences. If this is the case, implicit negation would result in greater activation of the sensory-motor system (see Egorova et al., 2013 for ERP data on semantic and pragmatic processing; see Kuberberg et al., 2000 for fMRI results on the neural correlates of semantic and pragmatic processing). Due to the limited literature on this topic, we decided to adopt an exploratory approach. Therefore, another alternative scenario should be also considered. Since implicit negative sentences do not have an explicit syntactic marker for negation, they might be processed in a similar way to affirmatives sentences and thus they may not involve the motor inhibitory mechanisms. However, given our pragmatic and inferential view of language, we consider this scenario to be the less likely.

## Experiment 1

### Materials and methods

#### Participants

The sample size was established a priori using statistical power analysis (a priori sample sizeevaluated for F-test for a within repeated measures ANOVA: *α* = 0.05, *1 - β* = 0.80, effect size = 0.15, one group, 6 measurements, correlation among rep measures = 0.3 and non-sphericity correction = 0.7; G*Power 3.1.9.4; Faul et al., 2009). Since the current literature on the Go/No-go task regarding the involvement of inhibitory mechanisms in sentence negation processing found no behavioral results and did not investigate the implicit forms of negations, we considered a small effect size. The analysis yielded a minimum sample size of 86 participants. We recruited 109 healthy young adults (56 females, Mean ± Standard Deviation (M ± SD) age = 25.29 ± 4.40, range = 18–35) to take part in the study. Inclusion criteria included (i) age range from 18 to 35 years old; (ii) right-hand dominance (Oldfield, 1971); (iii) Italian native speakers; (iv) absence of learning disabilities or other language impairments; and (v) normal or corrected-to-normal visual acuity. Since we conducted the study online due to the outbreak of the COVID-19 pandemic, we needed to ensure accurate and homogeneous timing of both stimulus presentation and responses. The Labvanced software (version 2.10; Finger et al., 2017; https://www.labvanced.com/), used to carry out this study, assessed these aspects for the entire duration of the experimental session in terms of median and SD of the delay. We excluded participants with a median offset greater than or equal to 20 ms and with a SD greater than or equal to 12 ms. Therefore, we discarded 9 participants from the data analysis. Additionally, we excluded 14 participants due to poor performance in the recognition task (mean accuracy < 75%; see below). In conclusion, our final sample consisted of 86 healthy participants (44 females; *M* ± *SD* age = 24.97 ± 4.17 years, range = 18–35; Education = 35 high school, 28 bachelor, 16 master, and 7 post-graduate).

All participants provided written informed consent to participate in the study, which was approved by the ethical committee of the Department of Cognitive Sciences, Psychology, Education and Cultural Studies, University of Messina (protocol number: COSPECS_7_2021) and conducted in accordance with the Declaration of Helsinki (2013).

#### Stimuli validation

The stimuli consisted of 15 two-word sentences, with five sentences for each of the three conditions: Affirmative, Explicit Negative, Implicit Negative (see Table 1 for the complete list of stimuli). The selection of stimuli was carried out through a validation procedure. The authors initially created a list of 21 verbs that could be considered as implicit negation, along with 11 filler verbs that did not express implicit negation. This list was then evaluated by a group of 20 participants (10 females; *M* ± *SD* age = 41.10 ± 14.37, range = 24–70; Education = 6 high school, 5 bachelor, 4 master, 5 post-graduate). The participants in the validation study did not take part in the subsequent experimental sessions. The validation study was administrated online using Psytoolkit (Stoet, 2010, 2017). For each verb, participants were asked to judge whether its meaning implied a negation. For example, when presented with the Italian verb “ignorare” (“to ignore”), participants were requested to judge if it was an implicit form of negation. If they answered positively, they were also asked to explicitly express the negative meaning of the verb in a lexical recall task. In this example, participants mostly defined “to ignore” as “non sapere” (“to not know”). This procedure served a dual purpose: to select the implicit negative verbs; and to select the verbs that were most frequently recalled by participants in the lexical recall task. These verbs were then used to build the Affirmative and Explicit Negative sentences that matched the Implicit Negative ones. Only verbs that were correctly recognized as expressing an implicit negation by 80% of participants were selected, resulting in a total of five Implicit Negative verbs and their respectively Explicit counterparts (see Table 1). The five selected Implicit Negative verbs and their counterparts were balanced for frequency of use (*t*(4) = -1.43; *p* = .23; Bambini & Trevisan, 2012), number of syllables (*t*(4) = 0.78; *p* = .48) and number of characters (*t*(4) = 0.53; *p* = .62).

Finally, these verbs were used to create 15 two-words sentences. The first-person pronoun was added to the Affirmative and Implicit Negative verbs, while the negative particle “non” (equivalent to “I don’t” in English) was added to the Explicit Negative verbs (e.g., Affirmative: “Io so/I know”, Explicit Negative sentence: “Non so/I don’t know”, Implicit Negative sentence: “Io ignoro/I ignore). It is important to emphasize that Italian is a pro-drop language, hence subject in explicitly negative sentences may be omitted. Thus, each sentence consisted of two words.

**Table 1:** – List of all the experimental stimuli. In *italics* the English translation of the sentence

Implicit Negative	Affirmative	Explicit Negative
Io digiuno *(I fast)*	Io mangio *(I eat)*	Non mangio *(I don’t eat)*
Io taccio *(I shut up)*	Io parlo *(I speak)*	Non parlo *(I don’t speak)*
Io rifiuto *(I refuse)*	Io accetto *(I accept)*	Non accetto *(I don’t accept)*
Io vieto *(I forbid)*	Io permetto *(I allow)*	Non permetto *(I don’t allow)*
Io ignoro *(I ignore)*	Io so *(I know*)	Non so *(I don’t know)*

#### Experimental procedure

**Fig. 1:**
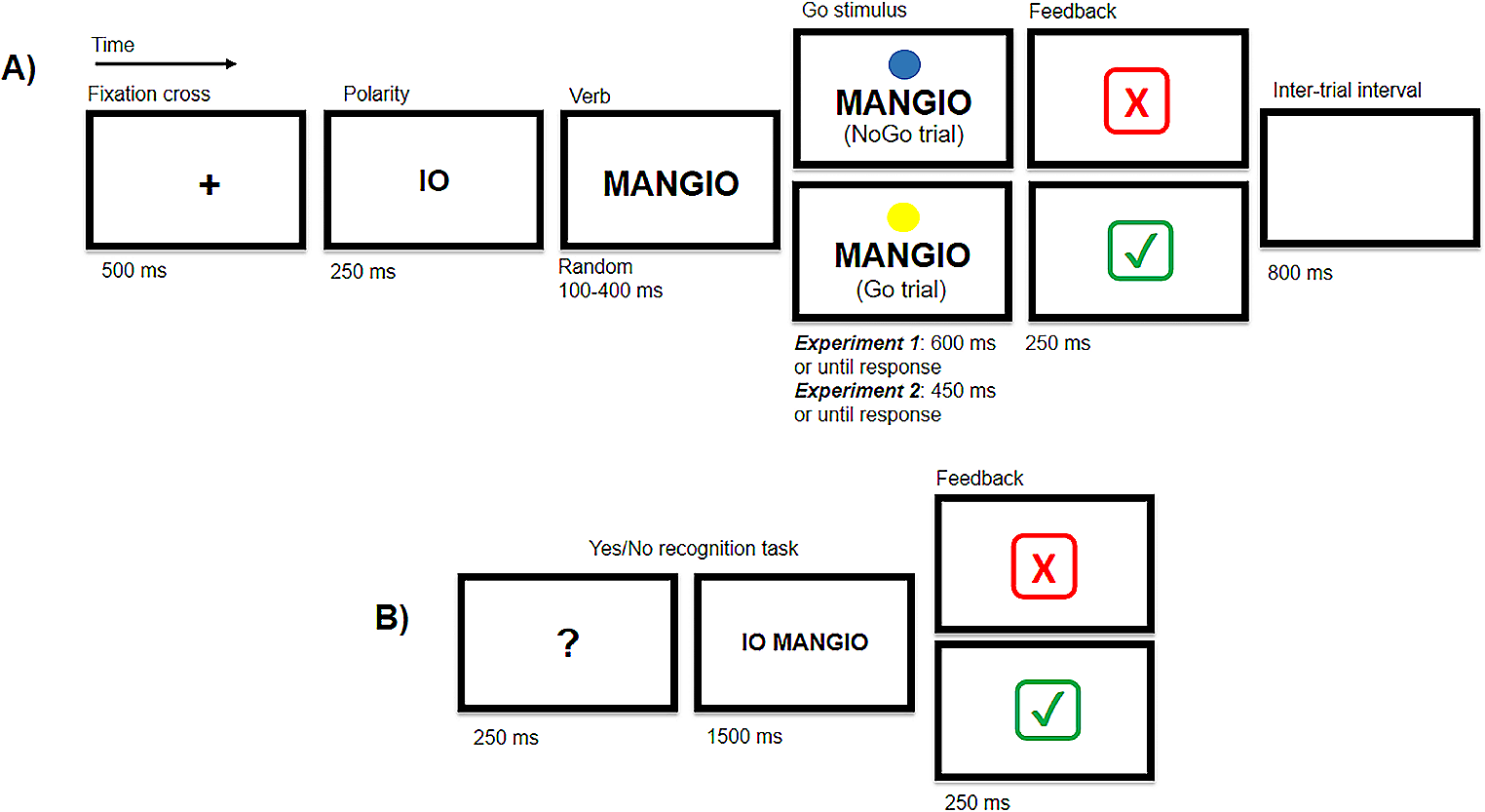
Experimental procedure. **(A)** The experimental trial. Italian two-words sentences were presented word by word at the center of the screen. Each trial started with a 500 ms fixation cross, followed by the sentence polarity-referred word (i.e., “Io/I” for affirmative and implicit negative sentences and “Non/I don’t” for explicit negative sentences) and subsequently followed by the verb. The duration of the solely verb presentation was randomized between 100 and 400 ms to prevent anticipatory responses. Participants were instructed to perform the Go/No-Go task by pressing the space bar when a yellow circle appeared above the verb, indicating a Go trial, and by withholding their response when the circle was blue, indicating a No-Go trial. Participant had 600 ms (Experimental 1) or 450 ms (Experimental 2) to respond to the go-signal. Performance was marked by visual feedback. Each trial ended with an inter-trial interval of 800 ms. **(B)** The recognition task. 11.85% of the total trials were followed by a yes/no recognition task. Participants were instructed to determine whether a two-word sentence was identical (in terms of both polarity and verb) or different (either in polarity or verb) from the sentence presented in the previous experimental trial. They had 1500 ms to press the mouse button when the sentences were identical. Participants’ performance was indicated by visual feedback

The sentences were presented word-by-word at the center of the screen, written in black capital letters (Arial font size 48 points) on a grey background. Each trial started with a 500 ms fixation cross, followed by the word related to the sentence polarity (i.e., “Io/I” for affirmative and implicit negative conditions and “Non/I don’t” for explicit negative condition) displayed for 250 ms, and subsequently followed by the verb. A colored circle appeared above the verb after a random delay between 100 and 400 ms. In Go trials, participants were instructed to respond as quickly and accurately as possible to the appearance of a yellow circle using the space bar with their right index, while in No-Go trials, marked by a blue circle, participants were instructed to withhold their response. Participants had 600 ms to respond to the circle; after this time, the answer was considered omitted. The corrected or erroneous responses were indicated by a 250 ms positive (green mark) or negative (red cross) visual feedback. An 800 ms inter-trial interval (ITI) concluded each trial (see Fig. 1, panel A). Catch trials were included to maintain participants’ attention and ensure sentence comprehension. In the catch trials (*N* = 32; 11.85% of the total trials) the ITI was followed by a yes/no recognition task, preceded by a 250 ms white question mark on a black background. In the catch trials, participants were required to determine whether a two-word sentence was identical or different (either in polarity or verb) from the one presented in the previous trial. Participants had 1500 ms to press the space bar. In 50% of recognition tasks, the sentences were “identical”, while in the remaining 50%, they were “different” (25% for polarity and 25% for verb). The correct or erroneous execution of the recognition task was marked by the same 250 ms positive or negative visual feedback (see Fig. 1 panel B).

Each sentence was repeated 18 times, resulting in a total of 270 trials. Among these, 33% were No-Go trials (6 trials for each experimental condition, for a total of 90 trials) and 67% were Go trials (12 trials for each experimental condition, for a total of 180 trials). The sentences were divided into three blocks of 90 trials, each lasting three minutes. Rest periods were allowed between blocks. In each block, the proportion between Go and No-Go trials was maintained at 2:1, respectively, and they were also balanced for condition (i.e., affirmative, explicit negative, implicit negative).

A training session consisting of 27 trials was conducted to familiarize participants with the experimental procedure. Among the 27 trials, six were followed by the recognition task. To avoid habituation, all the training trials included sentences that were not used in the experimental session.

At the end of the experiment, all the verbs used in the experimental stimuli were rated for arousal (“How intense do you judge the word?”) and valence (“How do you judge the valence of the word?”), using a Visual Analogue Scale, without any time constraints. Arousal ratings ranged from 0 = *very low* to 100 = *very high*, while valence ratings ranged from − 50 = *negative* to 50 = *positive.* It is important to specify that participants rated only the verb and not the whole sentence. Therefore, they rated a total of 10 verbs: the 5 verbs used to create affirmative and explicit negative sentences (i.e., mangio, parlo, accetto, permetto and so), and the five verbs used to construct the implicit negative sentences (i.e., digiuno, taccio, rifiuto, vieto e ignoro). The entire experimental paradigm was created and controlled using Labvanced software (version 2.10; Finger et al., 2017; https://www.labvanced.com).

#### Analysis

We considered mean RTs in Go trials and the percentage of commission errors (CoERs) in No-go trials, i.e., instances in which participants incorrectly responded to the No-go signal, as behavioural indexes of response inhibition proficiency. We excluded Go trials with RTs exceeding three standard deviations above and below the mean. Commission error rates were calculated by dividing the number of commission errors in a condition by the total number of trials in the same condition and multiplying it by 100.

Since all Go RTs variables were normally distributed [Shapiro-Wilk test (Affirmative: W = 0.99; *p* = .688; Explicit Negative: W = 0.98; *p* = .164; Implicit Negative: W = 0.98; *p* = .202)], we performed a one-way parametric ANOVA to compare mean Go RTs among the three conditions [within-participants factor: Polarity (3 levels: Affirmative, Explicit Negative, Implicit Negative)]. However, it is important to emphasize that the outbreak of the COVID-19 pandemic forced us to carry out the study in the online modality, making it impossible to control participants as in a laboratory setting. Hence, to control for participant’s variability, we chose to perform also a mixed-models analysis, including participants as random factor. Additionally, we included the stimulus as a random effect to enhance control over the observed effect. Following a hierarchical approach, we firstly created a simple model using only one parameter [Polarity] as independent fixed variable, and then we added Participants and Stimuli intercepts as random effects with the aim to evaluate whether its inclusion improved model fit. Likelihood ratio tests, Akaike Information Criterion (AIC) and Bayesan Information Criterion (BIC) were used to establish whether the inclusion of main effect and random effect would significantly improve model fit (for additional information regarding the selected model, please refer to Supplementary Table S1 available online). In this model, we controlled the presence of outliers by means of standardized model residuals and setting a threshold value for Cook’s distance (threshold = 1). However, no outliers were identified.

As for CoERs, participants made a low percentage of errors (Affirmative: *M* ± *SD* = 2.29 ± 3.48; Explicit Negative: *M* ± *SD* = 1.70 ± 3.30; Implicit Negative: *M* ± *SD* = 1.67 ± 2.69), hence we employed the Brown–Forsythe F star test that allows controlling the floor effect (DACF package for R; Liu & Wang, 2021) [within-participants factor: Polarity (3 levels: Affirmative, Explicit Negative, Implicit Negative)].

Lastly, we compared participants’ ratings for valence and arousal of the only verbs [within-participants factor: Polarity (2 levels: Affirmative/Explicit Negative, Implicit Negative)]. Valence ratings were analysed using a parametric paired *t*-test [Shapiro-Wilk test (Affirmative/Explicit Negative: W = 0.98; *p* = .204; Implicit Negative: W = 0.98; *p* = .456)], while Arousal ones were compared using a Wilcoxon test [Shapiro-Wilk test (Affirmative/Explicit Negative: W = 0.97; *p* = .096; Implicit Negative: W = 0.91; *p* < .001)]. Bonferroni corrections were applied to all post hoc tests, and effect sizes were reported as partial eta-squared and Cohen’s d.

All analyses were performed using MATLAB (version 2021a) and R Studio software (version 4.0.0; R Core Team, 2020). The datasets analysed during the current study are available in the OSF repository, https://osf.io/v8rf9/?view_only=113f8856407c42988785e8cbc94c7464.

## Results

### Go RTs – parametric ANOVA

**Fig. 2:**
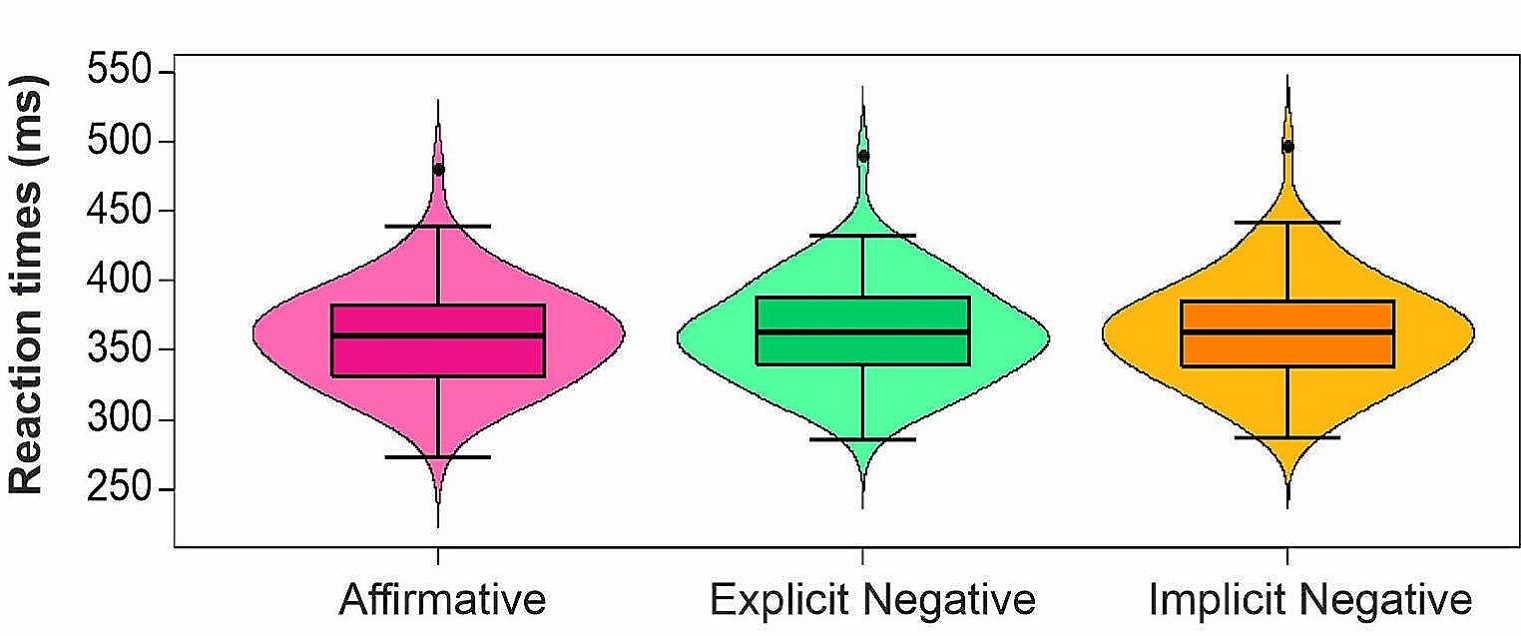
Effect of sentence polarity on mean Go RTs. The parametric ANOVA revealed a significant difference between Affirmative and Implicit Negative RTs. Moreover, taking into consideration Participants and Stimuli as random effects in a mixed model analysis, we also found a significant difference in RTs between Affirmative and Explicit Negative conditions. The lower and upper box plot’s boundary indicates the first and third quartile, respectively, the median is marked with a black line and error bars represents standard errors (SEs) of the means. The width of the violin plots depicts kernel probability density, i.e., the relative frequency of the data. The graph was created from raw means

The one-way ANOVA on mean Go trials RTs (Fig. 2) showed a significant main effect of Polarity (F(1.93, 164.40) = 4.51, PES = 0.05, *p* = .013). Post hoc comparison revealed that RTs were significantly longer for Implicit Negative sentences (*M* ± *SD* = 363.42 ± 37.97 ms) compared to the Affirmative ones (*M* ± *SD* = 359.97 ± 36.09 ms; *t*(85) = -2.63, *p* = .030; Cohen’s *d* = -0.24, 95% Confidence Interval/CI = [-6.06; -0.84]). Explicit Negative sentences did not differ from both Affirmative and Implicit Negative conditions (*M* ± *SD* = 362.73 ± 36.08; all *p*_s_ > 0.05).

### Go RTs – linear mixed model

The model explained 32% of the variance of RTs considering both Participants and Stimuli as random effects (*R*^*2*^*m* = 0.0005; *R*^*2*^*c* = 0.32). The model showed a significant main effect of Polarity (*χ*^*2*^(2) = 12.00, *p* = .002), highlighting faster RTs in Affirmative sentences than both Explicit Negative (*t*(132)= -2.47, *p* = .04) and Implicit Negative conditions (*t*(132)= -2.47, *p* = .04).

### Commission error rates

The Brown–Forsythe F star test applied to commission error rates did not show any significant effect (F = 3.31, *p* = .07).

### Valence and arousal

The paired *t*-test on Valence ratings showed a significant difference (*t*(85) = 23.80; *p* < .0001), given that Implicit Negative verbs (*M* ± *SD* = -24.03 ± 12.98) were rated as more negative than Affirmative/Explicit Negative ones (*M* ± *SD* = 21.74 ± 10.11).

The Wilcoxon test on Arousal ratings showed a significant difference (V = 1012; *p* < .001): participants attributed greater arousal to Implicit Negative verbs (*M* ± *SD* = 68.79 ± 18.08) than Affirmative/Explicit Negative ones (*M* ± *SD* = 61.92 ± 11.00).

## Experiment 2

Previous studies have shown that action-related and non-action related words are semantically processed at different time points, with action-related words being processed around 200 ms, and non-action related words around 250 ms from stimulus onset (Papitto et al., 2021). Negation, being an abstract and non-action related word, falls into the latter category. Given these findings, the timing of the response time-window to the Go stimulus becomes a crucial factor in understanding the interaction between the processing of linguistic negation and the recruitment of motor inhibitory mechanisms. In the first experiment, we aimed to investigate this interaction by examining the involvement of motor response inhibition resources during the processing of both explicit and implicit negative sentences. However, the 600 ms time-window we initially employed might be too long to capture the nuances of this interaction. This might be particularly true considering the poor amount of CoERs made by participants. Therefore, we carried out a second study, in which we reduced the time-window to respond to the Go stimulus from 600 ms to 450 ms. The shortening of the response time-window should induce greater sense of time pressure in the participants, which could lead to higher CoERs. This change of the experimental design could enable a better understanding of the temporal dynamics of the motor inhibitory processes involved in the processing of explicit and implicit negative sentences.

## Materials and methods

### Participants

We considered the same sample size estimation (i.e., 86 participants), and inclusion and exclusion criteria used in experiment 1 (see paragraph 2.1). 103 healthy young adults (48 females, *M* ± *SD* age = 24.42 ± 3.91 years, range = 18–35) took part in the study. Two participants were discarded due to a poor-quality offset, while other 14 were excluded due toa poor performance in the recognition task (mean accuracy < 75%). Thus, our final sample was composed by 87 healthy participants (44 female; *M* ± *SD* age = 24.43 ± 3.91, range = 18–35; Education = 43 high school, 24 bachelor, 17 master, and 3 post-graduate). All participants provided written informed consent to participate in the study, which was approved by the ethical committee of the Department of Cognitive Sciences, Psychology, Education and Cultural Studies, University of Messina (protocol number: COSPECS_7_2021) and conducted in accordance with the Declaration of Helsinki (2013).

### Stimuli and experimental design

We used the same stimuli and experimental design of experiment 1, with the only difference that the time to respond to the go-stimulus, was reduced from 600 ms to 450 ms.

### Analysis

Analyses were performed using the same approach of experiment 1 (see paragraph 2.4.). However, since mean Go RTs were not normally distributed [Shapiro-Wilk test (Affirmative: W = 0.96; *p* = .018; Explicit Negative: W = 0.97; *p* = .041; Implicit Negative: W = 0.96; *p* = .012)], we used the non-parametric Friedman test. Moreover, we used the generalized linear mixed-models analysis to control the variability given by participants and stimuli, included as random factors (see Supplementary Table S2 online for more details about the selected model). No outlier was identified and excluded. Regarding CoERs, the attempt to shorten the response-window to make the task more difficult still resulted in a low frequency of such errors (Affirmative: *M* ± *SD* = 2.76 ± 3.14; Explicit Negation: *M* ± *SD* = 2.53 ± 3.63; Implicit Negation: *M* ± *SD* = 2.15 ± 3.05). Thus, CoERs were analysed using the same Brown–Forsythe F star test of the experiment 1. Lastly, valence and arousal ratings were analysed using a parametric and a non-parametric t-test, respectively [Shapiro-Wilk test: Valence (Affirmative/Explicit Negative: W = 0.98; *p* = .325; Implicit Negative: W = 0.98; *p* = .370); Arousal (Affirmative/Explicit Negative: W = 0.97; *p* = .023; Implicit Negative: W = 0.97; *p* = .027)].

## Results

### Go RTs – non parametric ANOVA

**Fig. 3:**
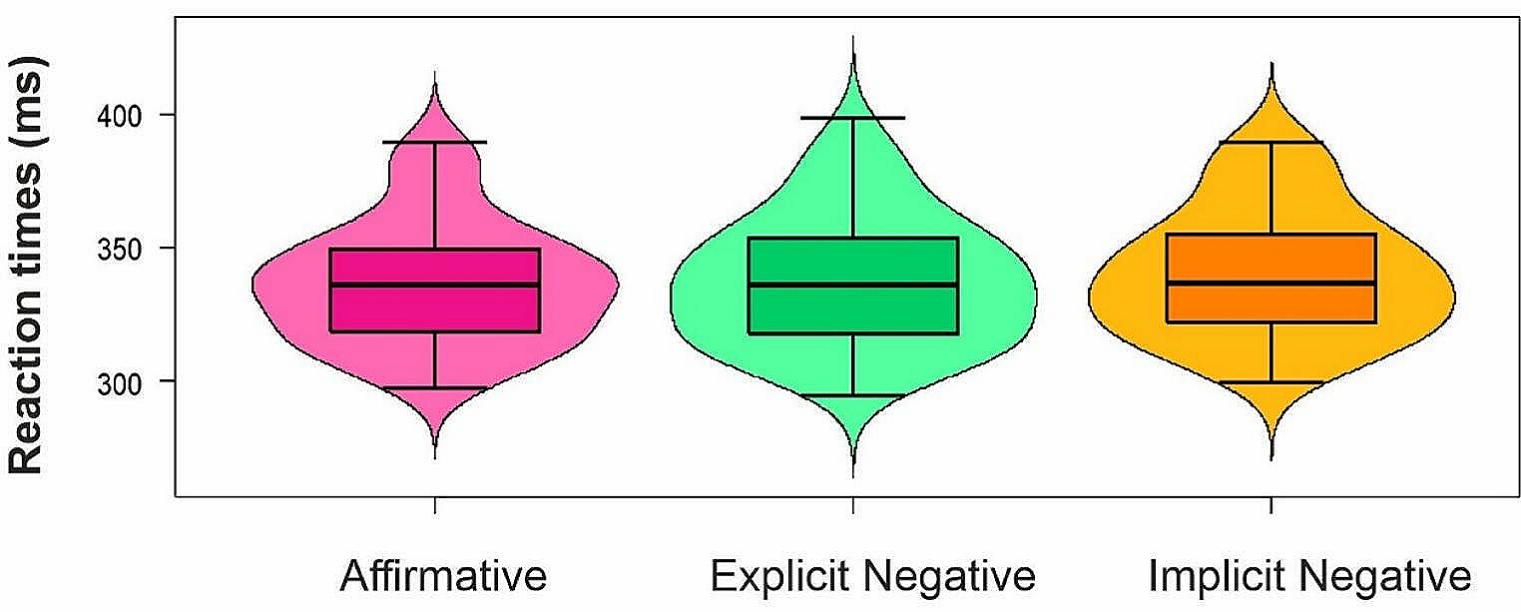
Effect of sentence polarity on mean Go RTs. The non-parametric ANOVA revealed a significant difference between Affirmative and Implicit Negative RTs. Moreover, taking into consideration Participants and Stimuli as random effects in a mixed model analysis, we also found a significant difference in RTs between Explicit Negative and Implicit Negative conditions. The lower and upper box plot’s boundary indicates the first and third quartile, respectively, the median is marked with a black line and error bars represent standard errors (SEs) of the means. The width of the violin plots depicts kernel probability density, i.e., the relative frequency of the data. The graph was created from raw means

The Friedman test on mean Go trials RTs (Fig. 3) showed a significant main effect of Polarity (χ2(2) = 13.52; PES = 0.08; *p* = .001). Post hoc comparison revealed that RTs were significantly longer for Implicit Negative sentences (*M* ± *SD* = 340.11 ± 23.54 ms) compared to the Affirmative ones (*M* ± *SD* = 336.25 ± 22.77 ms; *p* = .001; Cohen’s *d* = -0.50; 95% Confidence Interval/CI = [-5.49; -2.24]). Explicit Negative sentences (*M* ± *SD* = 337.54 ± 24.26) did not differ from both Affirmative (*p* = .522) and Implicit Negative conditions (*p* > .07).

### Go RTs – linear mixed model

The model explained 25% of the variance in RTs considering Participants and Stimuli as random effects (*R*^*2*^*m* = 0.001; *R*^*2*^*c* = 0.25). The model showed a significant main effect of Polarity (*χ*^*2*^(2) = 23.45; *p* < .0001), highlighting longer RTs for Implicit Negative sentences compared to both Affirmative (*z* = -4.77, *SE* = 0.80, *p* < .0001) and Explicit Negative conditions (*z* = -3.13, *SE* = 0.80, *p* = .005).

### Commission error rates

The Brown–Forsythe F star test applied to commission error rates did not show any significant effect (F = 3.77, *p* = .054).

### Valence and arousal

As for valence ratings, the parametric *t*-test revealed that participants judged Affirmative/Explicit Negative verbs (*M* ± *SD* = 20.82 ± 9.19) as significantly more positive compared to Implicit Negative ones (*M* ± *SD* = -24.43 ± 12.16; *t*(86) = 23.10; *p* < .0001).

The non-parametric Wilcoxon test on arousal ratings showed that Implicit Negative verbs (*M* ± *SD* = 70.89 ± 14.16) were rated as more arousing compared to the Affirmative/Explicit Negative counterpart (*M* ± *SD* = 61.72 ± 12.37; *V* = 939.5; *p* < .0001).

## Arousal and valence assessment: a follow-up survey

The ratings of arousal and valence in the previous experiments were carried out using verbs in the infinitive mood. We asked participants to rate arousal and valence of the verbs used in the Affirmative and Explicit Negative sentences (e.g., “to know”) on the one hand, and the ones used in the Implicit Negative sentences on the other hand (e.g., “to ignore”). However, this procedure didn’t allow us to compare arousal and valence in the two different types of negation. To better address this issue, using an online survey, we asked an independent sample of participants to rate the arousal and valence of the 15 experimental whole sentences (e.g., “I know”, “I don’t know”; “I ignore”).

## Materials and methods

### Participants

29 participants (16 females, *M* ± *SD* age = 30.14 ± 8.24, range = 21–58; Education = 8 high school, 6 bachelor, 10 master, 5 post-graduate) took part in the study. All participants provided written informed consent to participate in the study, which was approved by the ethical committee of the Department of Cognitive Sciences, Psychology, Education and Cultural Studies, University of Messina (protocol number: COSPECS_7_2021) and conducted in accordance with the Declaration of Helsinki (2013).

### Method

Participants were instructed to rate valence and arousal of the same 15 sentences, used in the previous two experiments. Sentences were randomly presented at the centre of the screen two times, one for each of the following questions: (i) valence: “*How would you judge the emotional valence expressed by the sentence?*”; (ii) arousal: “*How would you judge the emotional intensity expressed by the sentence?*”. Participants were asked to answer to the question using a VAS without any time constraints. Valence ranged from − 50 = *negative* to 50 = *positive*, the central point corresponded to *neutral*. Arousal ranged from 0 = *very few* to 100 = *very much*. Participants were informed that they could respond at any point on the bar. The questionnaire was created using Psytoolkit (Stoet, 2010, 2017) and online administrated.

### Analysis

For both valence and arousal, after outliers’ exclusion, variables were normally distributed. Thus, we performed two parametric ANOVAs. Post hoc comparisons were Bonferroni corrected, and effect sizes were reported as partial eta-squared and Cohen’s d. Analyses were performed using R Studio software (version 1.4.1717; R Core Team, 2019).

## Results

For the valence rating, the ANOVA showed a significant main effect of Condition (*F*(1.38, 38.64) = 148.51; Effect Size = 0.84; *p* < .001) indicating that all the experimental conditions differed from each other. Affirmative sentences (*M* ± *SD* = 16.30 ± 8.91) were rated more positive than both Explicit Negative (*M* ± *SD* = -18.74 ± 11.29; *CIs* = 28.85, 41.23; *p* < .0001) and Implicit negative sentences (*M* ± *SD* = -23.34 ± 9.66; *CIs* = 33.96, 45.33; *p* < .0001). Moreover, the Explicit Negative sentences were rated as more positive than Implicit Negative ones (*CIs* = 1.59, 7.62; *p* = .01). For the arousal rating, the ANOVA didn’t show significant effects (*F*(1.67, 46.66) = 2.86; Effect Size = 0.09; *p* = .076).

## Discussion

The purpose of this study was to examine the role of motor inhibitory mechanisms in the processing of linguistic implicit negation. Implicit negation refers to a form of negation that is not asserted through morphosyntactic elements or other expressions, which, through entailments, determine the representation of negative meaning in the minimal sentence. Thus, implicit negation is only present in the intended meaning of a sentence, and it relies on presuppositions or implicatures. So far, previous studies that have dealt with sentence negation under the Neural Reuse Hypothesis (Beltrán et al., 2021) have only investigated explicit forms of negation (Beltrán et al., 2018, 2019; De Vega et al., 2016; Foroni & Semin, 2013; García-Marco et al., 2019; Liu et al., 2019, 2020; Montalti et al., 2021; Papeo et al., 2016; Vitale et al., 2022). Hence, this study aimed to fill this gap. Two online experiments were conducted using a Go/No-Go paradigm.

Results from the ANOVA from both Experiment 1 and Experiment 2 revealed a significant difference between RTs in Affirmative and Implicit Negative conditions, with faster RTs for the Affirmative condition. However, no significant difference was found between Explicit Negation and the other two conditions. This lack of significant difference between Affirmative and Explicit Negative sentences aligns with previous Go/No-Go studies carried out by Beltran and colleagues, where a significant difference has not been found (Beltrán et al., 2019; De Vega et al., 2016; Liu et al., 2019, 2020). In addition, considering that the two experiments were carried out online, we decided to also control the variability given by participants and stimuli using a linear mixed model. In both studies (Experiment 1 and Experiment 2), such model confirmed a difference between Affirmative and Implicit Negative sentences and allowed to reveal other interesting results. Specifically, in Experiment 1 (longer time window for the response), we also found a significant difference between Affirmative and Explicit negative sentences, while the two negative conditions (Explicit negation and Implicit negation) did not differ. Differently, in Experiment 2 (shorter time window for the response) we found a significant difference between Explicit and Implicit Negative sentences, but not between Affirmative and Explicit Negative sentences. The output of mixed model analysis, together with the ANOVA, seems to suggest that the processing of Implicit Negation determines a stronger modulation of the mechanism for motor response inhibition compared to both Affirmative and Explicit Negative sentences.

Valence and arousal ratings assessed in the follow-up validation study on whole sentences (“I know”, “I don’t know”; “I ignore”) revealed significant results only for valence, with Implicit Negative sentences considered more negative than the other two experimental conditions, while no differences were found for arousal ratings.

Overall, based on our findings, we might assume that there is a gradient in the processing of linguistic negation, where Implicit Negation recruits the inhibitory mechanisms to a greater extent compared to its Explicit forms. Implicit Negation, having an inferential nature, may determine a deeper processing of the negative meaning compared to a likely shallow processing of explicit negation, thus leading to greater activation of the sensory-motor system (Egorova et al., 2013; Kuberberg et al., 2000).

We believe that incorporating Implicit Negation in our experimental design led us to unveil differences never appreciated in the literature. However, our pattern of results, although promising, is still partly unclear and many aspects need to be further investigated. In fact, in the mixed model analysis of Experiment 1, showed that Explicit Negation differed from Affirmative sentences, but not from the Implicit Negation. The opposite pattern was found in Experiment 2 (i.e., Explicit Negation differed from the Implicit one, but not from Affirmative sentences). The reason might be found in the time-window given to participants to respond (longer in Experiment 1, and shorter in Experiment 2). In this regard, a comparison with previous Go/No-go studies is difficult, as they never clearly reported the duration of the response time-window. Yet, little is known about the temporal activation of the inhibitory system when the latter is modulated by the processing of linguistic materials.

Notably, we must also acknowledge that in these kinds of experimental designs the recruitment of motor inhibitory resources is doubly modulated. On the one hand, by the No-Go trials as the motor inhibitory system is strongly activated by a successful response inhibition. On the other, by the processing of linguistic negation that according to the Neural Reuse Hypothesis (Beltran et al., 2021) recruits motor inhibitory resources. In the latter case, a weaker involvement of motor inhibitory mechanisms could be expected since it is activated only by linguistic materials. This double modulation might have contributed to determine this pattern of results.

Several aspects allow us to conclude that Implicit Negative sentences recruits motor inhibitory resources and that they do so stronger compared to Explicit Negative ones, ruling out other alternative explanations: 1) stimuli were balanced for frequency of use, and number of syllables and characters; 2) sentences were very easy to comprehend, excluding a difference in cognitive load. In fact, the selected verbs were identified as implicit negation verbs by 80% of the participants in the validation study; 3) the experimental task was simple, as suggested by the low amount of commission errors found in all experimental conditions; 4) valence ratings differed across the three experimental conditions, but these differences cannot explain our findings by themselves. Despite no behavioural difference has ever been found between Affirmative and Explicit Negative sentences, we might reasonably suppose that the former ones are always more positively valenced compared to the latter ones, as it was in our study. Conversely, whether the effect was driven by the stimuli’ valence, a difference between Affirmative and Explicit Negative sentences should have been found; 5) the experimental conditions did not differ for arousal. Thus, differences in our experimental condition likely depends solely on the level at which negation is processed.

The main limitation of our study is that it was done online, and therefore, in the future, it will be worthwhile to verify the reproducibility of the results in the laboratory. Moreover, the use of just five verbs for each condition might have influenced our findings, given that repetition can impact semantic comprehension. However, our rigorous initial validation guided us to include only five verbs. Expanding the variety of stimuli in future studies could help address this limitation.

Furthermore, future studies should implement experimental designs which allow to take into account the double modulation of the inhibitory mechanisms, and explore the link between such inhibitory resources and the implicit negation processing at a more functional level. As demonstrated in previous studies (Beltrán et al., 2019; De Vega et al., 2016; Liu et al., 2019, 2020), the EEG technique is a great tool to address this issue, allowing a deep assessment of the amplitude modulation of ERP components of interest. These limitations notwithstanding, our findings allow us to provide two main theoretical implications. Firstly, they provide further evidence that even abstract aspects of language, such as linguistic negation, have a bodily grounding in the sensory-motor system. Secondly, and most importantly, they support the idea that implicit and inferential meaning (i.e., pragmatic information) are grounded too in the same mechanisms that integrate action with perception. The latter point is particularly relevant since it expands our knowledge of the embodied grounding of the pragmatic account of language. Studying the involvement of the sensory-motor system in the processing of implicit, inferential and usage-based linguistic meaning represents frontline research for any embodied account of language. The study of the embodiment of the pragmatic aspects of language is certainly one of the most intriguing and cutting-edge line of research in the embodied cognition framework.

### Electronic supplementary material

Below is the link to the electronic supplementary material.

Supplementary Material 1

## Data Availability

The datasets analysed during the current study are available in the OSF repository, https://osf.io/v8rf9/?view_only=113f8856407c42988785e8cbc94c7464.

## References

[CR1] Alemanno F, Houdayer E, Cursi M, Velikova S, Tettamanti M, Comi G, Cappa SF, Leocani L (2012). Action-related semantic content and negation polarity modulate motor areas during sentence reading: An event-related desynchronization study. Brain Research.

[CR2] Aravena, P., Delevoye-Turrell, Y., Deprez, V., Cheylus, A., Paulignan, Y., Frak, V., & Nazir, T. (2012). Grip Force reveals the Context Sensitivity of Language-Induced Motor activity during action words Processing: Evidence from Sentential Negation. *Plos One*, *7*(12). 10.1371/journal.pone.0050287. 10.1371/journal.pone.0050287 PMC3515598 23227164

[CR3] Aron AR, Robbins TW, Poldrack RA (2014). Inhibition and the right inferior frontal cortex: One decade on. Trends in Cognitive Sciences.

[CR4] Bambini V, Trevisan M (2012). EsploraCoLFIS: Un’interfaccia web per le ricerche Sul Corpus E Lessico Di Frequenza dell’Italiano Scritto (CoLFIS). Quaderni Del Laboratorio Di Linguistica.

[CR5] Barsalou LW (2010). Grounded cognition: Past, Present, and Future. Topics in Cognitive Science.

[CR6] Bartoli E, Tettamanti A, Farronato P, Caporizzo A, Moro A, Gatti R, Perani D, Tettamanti M (2013). The disembodiment effect of negation: Negating action-related sentences attenuates their interference on congruent upper limb movements. Journal of Neurophysiology.

[CR8] Beltrán D, Muñetón-Ayala M, de Vega M (2018). Sentential negation modulates inhibition in a stop-signal task. Evidence from behavioral and ERP data. Neuropsychologia.

[CR7] Beltrán D, Morera Y, García-Marco E, De Vega M (2019). Brain inhibitory mechanisms are involved in the processing of sentential negation, regardless of its content. Evidence from EEG theta and beta rhythms. Frontiers in Psychology.

[CR9] Beltrán D, Liu B, de Vega M (2021). Inhibitory mechanisms in the processing of negations: A neural reuse hypothesis. Journal of Psycholinguistic Research.

[CR10] Carapezza, M., & Cuccio, V. (2018). Abductive inferences in pragmatic processes. *Further advances in pragmatics and philosophy* (pp. 221–242). Springer.

[CR11] Carpenter PA, Just MA (1975). Sentence comprehension: A psycholinguistic processing model of verification. Psychological Review.

[CR12] Chase WG, Clark HH (1972). On the process of comparing sentences against pictures. Cognitive Psychology.

[CR13] Clark, H. H. (1976). *Semantics and comprehension*. Mouton.

[CR14] Cuccio V (2011). On negation. What do we need to say no?. Rivista Italiana Di Filosofia Del Linguaggio.

[CR15] Cuccio, V. (2012). Is embodiment all that we need? Insights from the Acquisition of Negation. *Biolinguistics*, *February*, 259–275. http://www.biolinguistics.eu/index.php/biolinguistics/article/view/250.

[CR17] Cuccio V, Gallese V (2018). A Peircean account of concepts: Grounding abstraction in phylogeny through a comparative neuroscientific perspective. Philosophical Transactions of the Royal Society B: Biological Sciences.

[CR16] Cuccio V, Ambrosecchia M, Ferri F, Carapezza M, Piparo F, Lo, Fogassi L, Gallese V (2014). How the context matters. Literal and figurative meaning in the embodied language paradigm. Plos One.

[CR18] De Vega M, Morera Y, León I, Beltrán D, Casado P, Martín-Loeches M (2016). Sentential negation might share neurophysiological mechanisms with action inhibition. Evidence from frontal theta rhythm. Journal of Neuroscience.

[CR19] Di Cesare G, Errante A, Marchi M, Cuccio V (2017). Language for action: Motor resonance during the processing of human and robotic voices. Brain and Cognition.

[CR20] Egorova N, Shtyrov Y, Pulvermuller F (2013). Early and parallel processing of pragmatic and semantic information in speech acts: Neurophysiological evidence. Frontiers in Human Neuroscience.

[CR21] Faul F, Erdfelder E, Buchner A, Lang AG (2009). Statistical power analyses using G*Power 3.1: Tests for correlation and regression analyses. Behavior Research Methods.

[CR22] Finger, H., Goeke, C., Diekamp, D., Standvoß, K., & König, P. (2017). *LabVanced: a unified JavaScript framework for online studies. In International Conference on Computational Social Science (Cologne)*. *July*.

[CR23] Fischer MH, Zwaan RA (2008). Embodied language: A review of the role of the motor system in language comprehension. Quarterly Journal of Experimental Psychology.

[CR24] Foroni F, Semin GR (2013). Comprehension of action negation involves inhibitory simulation. Frontiers in Human Neuroscience.

[CR25] Gallese V (2008). Mirror neurons and the social nature of language: The neural exploitation hypothesis. Social Neuroscience.

[CR27] Gallese V, Cuccio V (2018). The neural exploitation hypothesis and its implications for an embodied approach to language and cognition: Insights from the study of action verbs processing and motor disorders in Parkinson’s disease. Cortex; a Journal Devoted to the Study of the Nervous System and Behavior.

[CR26] Gallese V, Lakoff G (2005). The brain’s concepts: The role of the sensory-motor system in reason and Language. Cognitive Neuropsychology.

[CR28] García-Marco, E., Morera, Y., Beltrán, D., de Vega, M., Herrera, E., Sedeño, L., Ibáñez, A., & García, A. M. (2019). Negation markers inhibit motor routines during typing of manual action verbs. *Cognition*, *182*(June 2018), 286–293. 10.1016/j.cognition.2018.10.020Glenberg A., and Gallese V. (2012). Action-based language: A theory of language acquisition production and comprehension. *Cortex*, 48, 905–922. 10.1016/j.cognition.2018.10.020 30390568

[CR29] Glenberg AM, Witt JK, Metcalfe J (2013). From the Revolution to Embodiment: 25 years of cognitive psychology. Perspectives on Psychological Science.

[CR30] Grice, H. P. (1989). *Studies in the way of words*. Harvard University Press.

[CR32] Horn, L. (1989). A natural history of negation.

[CR31] Horn LR (1996). Exclusive company: Only and the dynamics of vertical inference. Journal of Semantics.

[CR33] Horn, L. R. (2001). Flaubert triggers, squatitive negation, and other quirks of grammar. *Perspectives on Negation and Polarity Items*, 173–200.

[CR34] Jirak D, Menz MM, Buccino G, Borghi AM, Binkofski F (2010). Grasping language - A short story on embodiment. Consciousness and Cognition.

[CR35] Jones S (1968). Instructions, self-instructions and performance. The Quarterly Journal of Experimental Psychology.

[CR36] Kaup B (2001). Negation and its impact on the accessibility of text information. Memory and Cognition.

[CR39] Kaup B, Zwaan RA (2003). Effects of negation and situational Presence on the accessibility of text information. Journal of Experimental Psychology: Learning Memory and Cognition.

[CR37] Kaup, B., Lüdtke, J., & Zwaan, R. (2005). Effects of negation, truth value, and delay on picture recognition after reading affirmative and negative sentences. *Proceedings of the 27th …*.

[CR38] Kaup B, Lüdtke J, Zwaan RA (2006). Processing negated sentences with contradictory predicates: Is a door that is not open mentally closed?. Journal of Pragmatics.

[CR40] Kuperberg GR, McGuire PK, Bullmore ET, Brammer MJ, Rabe-Hesketh S, Wright IC, David AS (2000). Common and distinct neural substrates for pragmatic, semantic, and syntactic processing of spoken sentences: An fMRI study. Journal of Cognitive Neuroscience.

[CR43] Liu Q, Wang L (2021). T-test and ANOVA for data with ceiling and/or floor effects. Behavior Research Methods.

[CR42] Liu B, Wang H, Beltrán D, Gu B, Liang T, Wang X, de Vega M (2019). The generalizability of inhibition-related processes in the comprehension of linguistic negation. ERP evidence from the Mandarin language. Language Cognition and Neuroscience.

[CR41] Liu B, Gu B, Beltrán D, Wang H, de Vega M (2020). Presetting an inhibitory state modifies the neural processing of negated action sentences. An ERP study. Brain and Cognition.

[CR44] Liuzza MT, Candidi M, Aglioti SM (2011). Do not resonate with actions: Sentence polarity modulates cortico-spinal excitability during action-related sentence reading. Plos One.

[CR45] Logan GD, Cowan WB, Davis KA (1984). On the ability to inhibit simple and choice reaction time responses: A model and a method. Journal of Experimental Psychology: Human Perception and Performance.

[CR47] MacDonald MC, Just MA (1989). Changes in activation levels with negation. Journal of Experimental Psychology: Learning Memory and Cognition.

[CR48] Marrero H, Yagual SN, Gámez E, Urrutia M, Díaz JM, Beltrán D (2020). Negation interacts with motivational direction in understanding action sentences. Plos One.

[CR49] Mirabella, G., Iaconelli, S., Spadacenta, S., Federico, P., & Gallese, V. (2012). Processing of hand-related verbs specifically affects the planning and execution of arm reaching movements. *Plos One*, *7*(4). 10.1371/journal.pone.0035403. 10.1371/journal.pone.0035403 PMC3335064 22536380

[CR50] Mirabella G, Del Signore S, Lakens D, Averna R, Penge R, Capozzi F (2017). Developmental coordination disorder affects the processing of action-related verbs. Frontiers in Human Neuroscience.

[CR51] Montalti M, Calbi M, Cuccio V, Umiltà MA, Gallese V (2021). Is motor inhibition involved in the processing of sentential negation? An assessment via the Stop-Signal Task. Psychological Research Psychologische Forschung.

[CR52] Oldfield RC (1971). The assessment and analysis of handedness: The Edinburgh inventory. Neuropsychologia.

[CR53] Orenes I, Beltrán D, Santamaría C (2014). How negation is understood: Evidence from the visual world paradigm. Journal of Memory and Language.

[CR54] Papeo L, Hochmann JR, Battelli L (2016). The default computation of negated meanings. Journal of Cognitive Neuroscience.

[CR55] Papitto G, Lugli L, Borghi AM, Pellicano A, Binkofski F (2021). Embodied negation and levels of concreteness: A TMS study on German and Italian language processing. Brain Research.

[CR56] Pulvermüller F, Moseley RL, Egorova N, Shebani Z, Boulenger V (2014). Motor cognition-motor semantics: Action perception theory of cognition and communication. Neuropsychologia.

[CR57] R Core Team (2019). R: A language and environment for statistical computing. *Vienna: R foundation for statistical computing* Available at: https://www.Rproject.org/.

[CR58] Spadacenta, S., Gallese, V., Fragola, M., & Mirabella, G. (2014). Modulation of arm reaching movements during processing of arm/hand-related action verbs with and without emotional connotation. *PLoS One. 2014 Aug 5*;9(8):e104349. 10.1371/journal.pone.0104349 PMC4122433 25093410

[CR59] Speranza JL, Horn LR (2010). A brief history of negation. Journal of Applied Logic.

[CR60] Stoet G (2010). PsyToolkit: A software package for programming psychological experiments using Linux. Behavior Research Methods.

[CR61] Stoet G (2017). PsyToolkit: A novel web-based method for running online questionnaires and reaction-time experiments. Teaching of Psychology.

[CR62] Tettamanti M, Manenti R, Della Rosa PA, Falini A, Perani D, Cappa SF, Moro A (2008). Negation in the brain: Modulating action representations. Neuroimage.

[CR63] Tomasino B, Weiss PH, Fink GR (2010). To move or not to move: Imperatives modulate action-related verb processing in the motor system. Neuroscience.

[CR65] Vitale F, Monti I, Padrón I, Avenanti A, de Vega M (2022). The neural inhibition network is causally involved in the disembodiment effect of linguistic negation. Cortex; a Journal Devoted to the Study of the Nervous System and Behavior.

[CR71] World Medical Association. (2013). World Medical Association Declaration of Helsinki ethical principles for medical research involving human subjects. *JAMA: Journal of the American Medical Association*, *310*(20), 2191–2194. 10.1001/jama.2013.281053. 10.1001/jama.2013.281053 24141714

[CR64] Xiang M, Grove J, Giannakidou A (2016). Semantic and pragmatic processes in the comprehension of negation: An event related potential study of negative polarity sensitivity. Journal of Neurolinguistics.

