# Supplementary material for: The role of motor inhibition in implicit negation processing: two Go/No-Go behavioral studies
Source: Psychol Res. 2024 Mar 14;88(4):1169–81. doi: 10.1007/s00426-024-01941-0 (PMC11143020; doi:10.1007/s00426-024-01941-0)
Supplement: Supplementary file 1 — Supplementary Material 1 [file 426_2024_1941_MOESM1_ESM.docx]

**Supplementary Materials**

**The role of motor inhibition in implicit negation processing: two Go/No-Go behavioral studies**

| **Experiment 1** | | | | | | | | |
| --- | --- | --- | --- | --- | --- | --- | --- | --- |
| ***lme4::lmer(RTs ~ Polarity + (1\|ID) + (1\|Stimuli), data = df)*** | | | | | | | | |
|  | ***df*** | **AIC** | **BIC** | **R^2^_M_** | **R^2^_C_** | **Log-Lik** | **X2** | ***p*-value** |
| **Reduced model (Null Model)** | 2 | 167609.4 | 167624.7 | - | - | -83803 | - | - |
| **Polarity** | 4 | 167606.3 | 167636.8 | 0.0005 | - | -83799 | 7.1303 | .028 |
| **+ Random Intercept**  **ID** | 5 | 162292.3 | 162330.4 | 0.0005 | 0.32 | -81141 | 5315.9973 | < .0001 |
| **+ Random Intercept**  **Stimuli** | 5 | 167562.9 | 167601 | 0.0005 | 0.005 | -83776 | 5270.6411 | < .0001 |
| **+ Random Intercept**  **ID**  **Stimuli** | 6 | 162229.5 | 162275.2 | 0.0005 | 0.32 | -81109 | 5335.4498 | < .0001 |

**Table S1. Detailed description of nested models for each measure.**

**Table S2. Detailed description of nested models for each measure.**

| **Experiment 2** | | | | | | | | |
| --- | --- | --- | --- | --- | --- | --- | --- | --- |
| ***lme4::glmer(RTs ~ Polarity + (1\|ID) + (1\|Stimuli), data = df, family = gaussian)*** | | | | | | | | |
|  | ***df*** | **AIC** | **BIC** | **R^2^_M_** | **R^2^_C_** | **Log-Lik** | **X2** | ***p*-value** |
| **Reduced model (Null Model)** | 2 | 155507.7 | 155522.9 | - | - | -77752 | - | - |
| **Polarity** | 4 | 155494.4 | 155524.8 | 0.0012 | - | -77743 | 17.326 | .0002 |
| **+ Random Intercept**  **ID** | 5 | 151799.8 | 151837.8 | 0.0012 | 0.24 | -75895 | 3696.602 | < .0001 |
| **+ Random Intercept**  **Stimuli** | 5 | 155444.7 | 155482.7 | 0.0012 | 0.006 | -77717 | 3644.914 | < .0001 |
| **+ Random Intercept**  **ID**  **Stimuli** | 6 | 151730.6 | 151776.2 | 0.0012 | 0.25 | -75859 | 3716.103 | < .0001 |
